# Phenformin as an Anticancer Agent: Challenges and Prospects

**DOI:** 10.3390/ijms20133316

**Published:** 2019-07-05

**Authors:** Mª Eugenia García Rubiño, Esmeralda Carrillo, Gloria Ruiz Alcalá, Alicia Domínguez-Martín, Juan A. Marchal, Houria Boulaiz

**Affiliations:** 1 Department of Physical Chemistry, Faculty of Pharmacy, University of Granada, 18071 Granada, Spain; 2 Biopathology and Regenerative Medicine Institute (IBIMER), Centre for Biomedical Research, University of Granada, E-18100 Granada, Spain; 3 Department of Human Anatomy and Embryology, Faculty of Medicine, University of Granada, E-18012 Granada, Spain; 4 Instituto de Investigación Biosanitaria de Granada (ibs. GRANADA), University Hospitals of Granada-University of Granada, 18012 Granada, Spain; 5 Fundamental Biology Service, Scientific Instrument Center, University of Granada, 18071 Granada, Spain; 6 Research Unit “Modeling Nature” (MNat), University of Granada, 18016 Granada, Spain; 7 Department of Inorganic Chemistry, Faculty of Pharmacy, University of Granada, 18071 Granada, Spain

**Keywords:** biguanides, phenformin, cancer, diabetes type 2, cancer stem cells

## Abstract

Currently, there is increasing evidence linking diabetes mellitus (especially type 2 diabetes mellitus) with carcinogenesis through various biological processes, such as fat-induced chronic inflammation, hyperglycemia, hyperinsulinemia, and angiogenesis. Chemotherapeutic agents are used in the treatment of cancer, but in most cases, patients develop resistance. Phenformin, an oral biguanide drug used to treat type 2 diabetes mellitus, was removed from the market due to a high risk of fatal lactic acidosis. However, it has been shown that phenformin is, with other biguanides, an authentic tumor disruptor, not only by the production of hypoglycemia due to caloric restriction through AMP-activated protein kinase with energy detection (AMPK) but also as a blocker of the mTOR regulatory complex. Moreover, the addition of phenformin eliminates resistance to antiangiogenic tyrosine kinase inhibitors (TKI), which prevent the uncontrolled metabolism of glucose in tumor cells. In this review, we evidence the great potential of phenformin as an anticancer agent. We thoroughly review its mechanism of action and clinical trial assays, specially focusing on current challenges and future perspectives of this promising drug.

## 1. Introduction

In 2011, it was estimated that one in three adults had obesity, which is, together with aging, one of the two main risk factors for the development of age-related diseases, such as cancer [1]. The increase of type 2 diabetes (due to the increase in obesity, decrease in physical activity, and an inadequate diet), implies a great social impact, becoming a chronic disease that represents a serious challenge for health systems worldwide [2]. Data from the World Health Organization (WHO) reports that by 2030, diabetes will become the seventh most frequent cause of death in the world [3]. Epidemiological evidence suggests an association between the incidence of cancer and type 2 diabetes mellitus, two diseases with a global impact [4,5,6]. Various causes are common in both diseases, due to high levels of C-peptide [7], insulin resistance, hyperactivation of IGF-1 receptors due to the mitogenic effect of insulin [8], leptin, low adiponectin, and a pro-inflammatory environment [9]. This association is argued by the fact that oral antidiabetics, such as phenformin and metformin, have therapeutic applications in various types of cancer. Based on this association, new antineoplastic therapies have been developed, targeting insulin and/or insulin growth factors (IGFs). Moreover, the latest findings on the capacity of the biguanides to inhibit cell growth and partly slow down the progression of neoplastic cells further supports the diabetes–cancer relationship by showing these drugs as a “pharmacological link” between both pathologies [6,9,10].

Hyperglycemia and hyperinsulinemia increase the production of reactive oxygen species, among other processes, accelerating cell aging and increasing the predisposition to cancer. In cancer patients, it is important to check the glycemic level in blood because an inadequate control of this is related to an increase in morbidity and mortality [11,12]. Both diseases mainly have in common hyperinsulinemia, chronic inflammation, and endoplasmic reticulum stress, amongst other factors [12]. Insulin resistance and consequent hyperinsulinemia can directly induce carcinogenesis through the insulin receptor or indirectly, when there is an increase in the levels of insulin growth factors (IGFs), sex steroid hormones, and inflammatory processes, among others [13,14]. Recent studies showed that patients treated with sulfonylureas or exogenous insulin have a higher risk of cancer. If treated with biguanides, the risk of suffering from this disease decreases [12].

## 2. Cancer and Diabetes Type 2: Common Risk Factors

There are several common risk factors in type 2 diabetes and cancer that show an association between both diseases. As examples, we could highlight metabolic syndrome and obesity, biological constraints such as insulin resistance and hyperinsulinemia, increased inflammation, endoplasmic reticulum stress, abnormal metabolic states, and dysregulation of autophagy [4,6,9,15].

In 2010, the American Diabetes Association and the American Cancer Society published a consensus, “Diabetes and Cancer”, that recommended screening diabetic patients for the detection of the disease, in addition to a rigorous choice of antidiabetic treatment for patients with a high risk of cancer or recurrent cancer. They also demonstrated epidemiological evidence that supported the association between diabetes (mainly type 2) and an increased risk in the development of colon-rectum, liver, pancreas, breast, endometrium, and bladder cancer, and a lower risk of developing prostate cancer. There doesn’t appear to be any association for lung cancer, and there is no conclusive evidence regarding non-Hodgkin’s lymphoma or kidney cancer [6,9].

### 2.1. Hyperinsulinemia and Insulin Resistance

Type 2 diabetes initially presents itself with hyperglycemia, and as a consequence, hyperinsulinemia occurs; if not controlled, a decrease in the number of hepatic insulin receptors is produced. Due to the increase in hepatic glycogenolysis and gluconeogenesis, glucose levels rise, accompanied by metabolic disorders. Diabetes falls within the metabolic syndrome generating a biochemical environment suitable for the development of tumors in diabetic patients [11,13,15,16].

Insulin resistance generates endogenous hyperinsulinemia, and also hyperglycemia, as well as low levels of adiponectin and high levels of leptin; it increases the bioavailability of sex hormones. As a consequence, the pro-inflammatory environment is maintained, chronically influencing the initiation, promotion, and progression of the tumor [6,9,17]. Insulin is the main anabolic hormone that controls blood glucose levels. It is produced by pancreatic ß cells and binds to their membrane receptors, consisting of two α subunits and two β subunits, to develop its physiological functions [18,19,20]. The insulin receptors are mainly found in the liver, adipose tissue, and skeletal muscle.

In order for type 1 insulin-like growth factor (IGF-1) to exert its mitogenic functions, it has to bind to its receptor (IGF-1R), which is present in tissues [21]. When activated, the receptor becomes auto/phosphorylated, in turn phosphorylating other proteins, which causes an amplification of the signaling cascade, causing a mitogenic effect through mitogen-activated protein kinase (MAPK).

By potentiating other growth factors, the mitogenic effect of IGF-1 on the level of apoptosis, angiogenesis, and increased cell size, is more potent than that of insulin [6,18,19,20,22].

Due to the great homology between the receptors (IR and IGF-1R) and their ligands (insulin, IGF-1 and IGF-2), insulin can bind to IGF-1R and exerts mitogenic effects. IGF-1/2 can also bind to the IR insulin receptor, and both receptors, IR and IGF-1R, can in turn form hybrid receptors, IR/IGF-1R (HR), that trigger an amplification of the intracellular signaling cascade [6,17,23]. In cancer and in diabetes mellitus type 2 (DM2), overexpression of these hormone receptors is observed in addition to IGF-1 and hyperinsulinemia [6], which activates anti-apoptotic and mitogenic routes. In fact, women with a high circulating rate of IGF-1 have a 28% higher risk of developing breast cancer compared to women with low concentrations of IGF-1 [24]. Therefore, hyperinsulinemia not only increases the risk of producing cancer by the effects of insulin as a mitogenic hormone, but also by the increased production of IGF-1 by chronic hyperinsulinemia and by induction in the formation of hybrid receptors, which amplifies the mitogenic signaling cascade. It should be noted that in tumors, insulin, rather than its action to capture glucose, has a mitogenic effect, since the tumor cells have an insulin-independent glucose uptake mechanism [24].

### 2.2. Metabolic Syndrome

Metabolic syndrome is a grouping of at least three of the following five disorders: fasting plasma glucose, abdominal obesity, high blood pressure, high triglycerides, and low levels of high density lipoprotein (HDL). Metabolic syndrome is associated with an increased risk of developing type 2 diabetes.

A multinational program initiated in 2006 studied the relationship between diabetes and cancer by investigating metabolic syndrome factors that are associated with the risk of cancer. The results showed a greater association of metabolic syndrome with a series of cancers, such as colon–rectum, gastric, breast, liver, endometrium, pancreas, and bladder [4,25,26].

Essentially, the tumor metabolism is characterized by the following:

(1) Low respiratory rate: conditions of hypoxia that lead to low local availability of oxygen and an anaerobic metabolism [27].

(2) High glycolysis rate: In the absence of oxygen (anaerobic conditions), oxidative phosphorylation (Krebs cycle in the mitochondria) is blocked and the ATP obtained is by glycolysis, with a much lower energy yield of ATP production than under aerobic conditions. In addition, tumor cells have a higher rate of replication than normal cells, glucose being their main source of energy, since hypermetabolism has a low energy yield. In addition, decoupling from the Krebs cycle can produce energy dissipation in the form of heat, therefore increasing body temperature [27].

Biguanides block the aerobic pathway, and cells get energy from the anaerobic pathway, so for the tumor cell to get the same amount of energy as from the aerobic pathway, it needs much more glucose to function, thus accelerating metabolism. However, certain types of tumors, due to uncontrolled multiplication, carry out “aerobic” glycolysis fermentation even in the presence of oxygen (Warburg effect) [6,10,17,25].

(3) Insulin resistance for healthy tissues: Diabetic decompensation develops due to lower and worse glucose uptake.

The biguanides decrease the production of glucose in the liver so that the concentration of this in blood also decreases. In general, although the biological mechanism of the biguanides at the molecular level is not known, they improve the sensitivity of tissues to insulin. Metformin began to differentiate itself from other antidiabetics, such as sulphonylureas or insulin, due to its ability to decrease blood glucose levels, since it prevents glucose from being absorbed at the intestinal level and decreases insulin peaks produced by the pancreas. It is an antihyperglycemic agent, without being a hypoglycemic agent [10].

(4) High lactate production: In many tumors, oxidative phosphorylation is blocked, and pyruvate, the final product of glycolysis, does not enter the Krebs cycle but is instead diverted towards glycolysis, producing excess lactate. A characteristic of advanced tumors, in addition to hypoxia and the aforementioned glycolysis, is the increase of intracellular protons due to the existence of a pH gradient because of intra- and extracellular gradient differences. So the tumor cells expel protons to the extracellular medium to thereby alkalize their cytosol, activating the proton transporters to compensate and regulate their intracellular pH. Consequently, due to the bad tamponade of excreted protons, an acid microenvironment is created. The problem is that lactic acid is produced from the metabolism [17,24]. The adiponectin produced by adipocytes increases insulin sensitivity, thus decreasing plasma glucose levels and also improving the sensitivity to fatty acids, reducing plasma triglycerides. Adipocytes also secrete pro-inflammatory cytokines that inhibit the synthesis of adiponectin. In type 2 diabetes and obesity, the levels of pro-inflammatory cytokines are increased; this inhibits the synthesis and plasma levels of adiponectin, thus decreasing its action to inhibit insulin resistance. So in type 2 diabetes and obesity, the inhibition of cell proliferation, apoptosis, and angiogenesis induced by adiponectin is diminished. There is a positive relationship between the decrease in the concentration of adiponectin levels, cancer, and metabolic syndrome [17,28].

### 2.3. Diabetes Type 2 Increases Risk of Cancer

It has been shown that the risk of breast cancer in people with type 2 diabetes increases by 20% compared to non-diabetics [6]. Aromatase is an enzyme that, starting from testosterone, is involved in estrogen biosynthesis. As mentioned previously in obesity and type 2 diabetes, there is an increase in adipose tissue that enhances the activity of aromatase and also estrogen levels, which increases the risk of breast and endometrial cancer. In addition, the IGF-1R receptor and the estrogen receptor could jointly activate the MAPK pathway that activates cell proliferation [17]. Moreover, meta-analytic studies show that testosterone levels are lower in type 2 diabetics, which is related to a lower risk of prostate cancer [17]. However, in diabetics the risk of mortality is increased, and they also present a worse diagnosis with this type of cancer. In these patients, early diagnosis of prostate cancer is hampered because blood levels of prostate-specific antigen (PSA) are low, due to insulin and IGF levels, despite tumor proliferation [6].

### 2.4. Biguanides Have a Protective Function on Cancer in Diabetic Patients

Biguanides influence the development of cancer, largely due to their effects on insulin levels and also on plasma glucose, which causes insulin resistance and hyperinsulinemia [10]. Biguanides increase insulin sensitivity by increasing affinity and also the amount of insulin receptors [6]. So drugs such as biguanides decrease insulin levels or improve their sensitivity, and the contrary happens with those drugs that increase insulin levels, such as exogenous insulin or sulfonylureas [24]. The biguanides seem to have a protective function on cancer in diabetic patients, as has been shown in vitro, that inhibits cell transformation, cell proliferation, stops the cell cycle, and induces cell death, specifically in tumor stem cells in different types of breast cancer [6].

## 3. Phenformin and Cancer

### 3.1. Phenformin: the Origin

**Figure 1:**
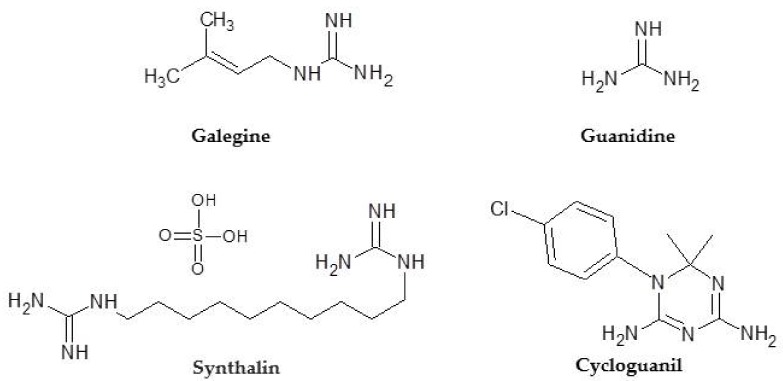
Compounds from which the chemical evolution of phenformin and buformide derives.

Biguanides are a family of compounds with different therapeutic applications. Studies conducted at the end of the 19th century indicated that, in addition to containing galegine (or isoprenyl guanidine), used for the treatment of type 2 diabetes (Figure 1); the flower of the *Galega officinalis* plant possessed a large quantity of guanidine, an organic compound found in urine. At the beginning of the 20th century, Watanabe studied the effects of guanidine (Figure 1) and demonstrated its hypoglycemic activity, as well as its toxicity [29]. Therefore, a series of guanidine analogues were synthesized to try to obtain less toxic derivatives with hypoglycemic properties. It was demonstrated that molecules with two guanidines in the same structure (biguanidines) had a greater hypoglycemic effect than those containing only one guanidine (monoguanidines). These results led to the multinational pharmaceutical company Schering developing Synthalin (Figure 1), since withdrawn from the market because of its toxicity.

**Figure 2:**
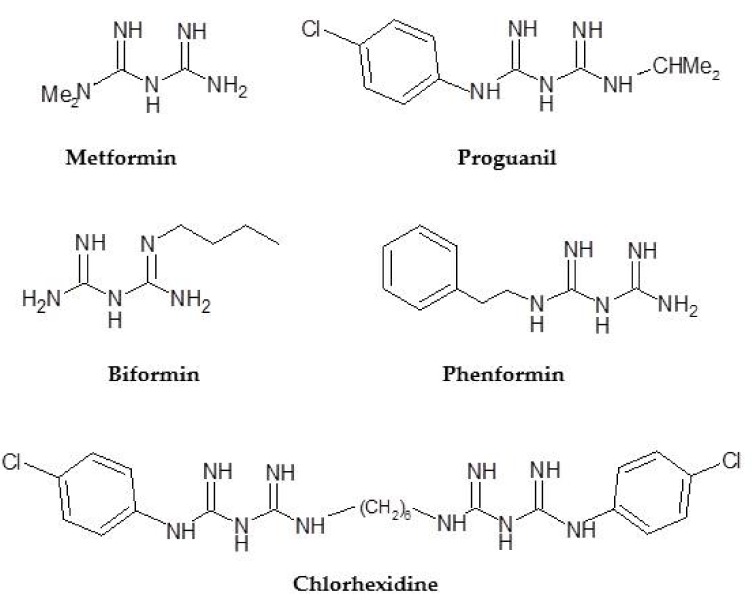
Biguanides with important medical activity.

At the same time, another group of researchers studied the biguanides and showed that they did not present toxicity in animals. However, in the middle of the 20th century, with the synthesis of metformin, the discovery of insulin as a hypoglycemic drug was made when metformin, the lead compound of biguanides, was tested in humans, not as a treatment for diabetes but to treat the symptoms of flu and malaria [30]. This research was conducted by Eusebio García, who discovered the antimalarial activity of metformin (and called it flumamin) due to its structural similarity to an antimalarial drug, chloroguanidine (proguanilil), whose active metabolite is cycloguanil (Figure 2) [30]. It was then tested, and in addition to reducing headaches and lowering the temperature, it decreased blood sugar levels. With the results of García, Sterne, a diabetes doctor who had worked with galegine and had checked the tolerance of metformin in animals, proposed taking metformin to the clinical stage and developing it under the name of Glucophage. Sterne’s work led, in the late 1950s, to the development of phenformin and buformin by Ciba-Geigy and Grünenthal [31,32]. But a decade later, buformin and phenformin were withdrawn from the market, in most countries towards the end of the 1970s [33,34], due to their lipophilic structure that gives them a high affinity for mitochondria membranes to interfere with oxidative phosphorylation and cause lactic acidosis [35,36].

The use of biguanides acquired a bad reputation amongst the scientific community; which is why metformin, 20 times less likely to cause lactic acidosis than the other two biguanides, entered the market 15 years later.

Currently, the commercially-available biguanide drugs are an oral antidiabetic compound, metformin, and an antimalarial agent, proguanil. Other important compounds in this series are chlorhexidine, a bactericidal and fungicidal agent, and biformin and phenformin, both antidiabetics withdrawn from the market in the late 1970s due to their high risk of fatal lactic acidosis (Figure 2). However, research on these drugs was still being carried out several years later.

### 3.2. Phenformin: Chemistry

**Figure 3:**
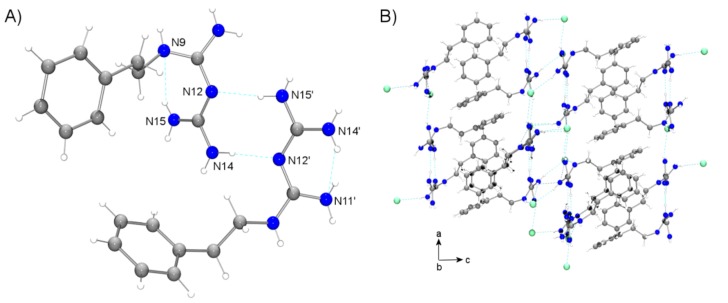
(**A**) H-bonded dimer of phenformin ligands within the crystal of phenformin hydrochloride [37], H-bonded as depicted as dashed cyan lines, and chloride ions have been omitted for clarity. (**B**) Crystal packing of phenformin hydrochloride crystal.

Despite the fact that phenformin was eventually withdrawn from the market in the late 1970s due to its high risk of fatal lactic acidosis, research on this drug was still being carried out several years later. For instance, the first crystal structure containing phenformin, as phenformin hydrochloride, was determined in 1979 by Herrnstad et al. [37]. In the crystal, two independent molecules co-exist in the asymmetric unit, both related by intermolecular H-bonds type N–H···N. However, limitations on X-ray crystallographic techniques at that time hampered the resolution of the protonation sites, which were tentatively located at the terminal imino group. This approximation was chemically based on (i) the observations within bond lengths in the structure, (ii) the relevant torsion angles, and (iii) the possibility of maximum delocalization of the C–N double bond and the allowance of positive charge, always in line with previously reported structures of the preferred tautomeric form of the unsubstituted biguanide [38] and its hydrochloride [39]. Moreover, the guanidine moiety did not show an overall planar conformation, with the phenyl group forming a dihedral angle of approximately 55° with respect to the biguanide moiety. In 1998, the crystal structure of phenformin hydrochloride was re-determined with higher resolution [40]. The presence of hydrogen bonded dimers by N–H···N interactions was confirmed, but this time the protonation site was identified. Note that protonation at N11 or N14 leads to the location of two H-atoms on both N11 and N14 (Figure 3A), with this structure being optimally stabilized by resonance. In addition, further details on the crystal packing were provided, such as two intramolecular interactions, further N–H···Cl intermolecular H-bonds, or C–H···π interactions (Figure 3).

Over the past decade, research on new antidiabetic drugs, as well as the tendency to investigate alternative biomedical applications of former drugs, has brought phenformin back to life. For instance, in an attempt to synthesize a zinc (II)-phenformin compound as a novel antidiabetic agent, the di-protonated form of phenformin was isolated, i.e., [40]. A drastic pH change is mainly responsible for this cationic form. Thus, phenformin exists in its monoprotonated form at pH ~7.2, while the di-protonated species requires pH ~2.0. Interestingly, this crystal structure resembles that of the aforementioned monoprotonated phenformin. In the asymmetric unit, pairs of two H-bonded independent N-phenethylbiguanidinium cations are found. Moreover, intramolecular N–H···N hydrogen bonds stabilize the cation conformation and intermolecular N–H···Cl bonds are involved in the crystal packing. The second protonation site is also located at one imino group (N12). This protonation directly affects the dihedral angle between the two guanidine moieties, which is smaller than other related monoprotonated biguanides. In addition, electron-π delocalization within the biguanide group has been evidenced by the average distance of all C–N bonds in the structure, i.e., shorter than single bonds but longer than double bonds. More recently, other compounds have been structurally characterized using phenformin and metformin cations as guest molecules of a family of water soluble calix(n) arenes [41].

### 3.3. Phenformin as Anticancer Agent

Today, there is enough evidence demonstrating that metformin, the most commonly prescribed drug for type II diabetes, has antitumor activity potential in a large variety of tumors [42]. Metformin has a well-established safety profile, and it has become clear that metformin has additional salutary effects, including anti-inflammatory, anti-aging, and antithrombotic properties. In an ongoing phase 1/2 clinical trial being carried out in the University of Pittsburgh Medical Center (Pittsburgh, PA, United States) the researchers are investigating the effects of metformin in a non-diabetic patient population. They hypothesize that metformin administration to non-diabetic adults will improve clinical outcomes to physiologic stress, by improving underlying immune and inflammatory responses, that can otherwise be deleterious (NCT03772964). In this study, subjects will provide both venous blood samples and stool samples, in addition to completing cognitive and physiologic testing at baseline, throughout a 90-day exposure to metformin and 30 days following exposure to metformin, in order to evaluate their immune, microbiome, cellular respiration, thrombotic, and inflammatory responses. We are sure that their results will be of great interest in elucidating the safety of metformin. Furthermore, several clinical trials using metformin, alone or in combination with other therapies, are underway in a variety of cancer types (NCT03137186; NCT02360618; NCT01941953; NCT03017833; NCT01930864). Moreover, promising results in pancreatic and endometrial cancer have been demonstrated [43,44,45]. As we mentioned above, phenformin is nearly 50 times as potent as metformin, but due to its association with a higher incidence of lactic acidosis, a major side effect of biguanides, it was withdrawn from clinical use, and consequently, the effect of phenformin on cancer has rarely been studied. In this context, the fact that treatment with phenformin can lead to lactic acidosis while metformin does not [46] suggests that these two biguanides act through different pathways. Hence, arguments that phenformin could, with distinct dosing and usage, be a promising anticancer agent has aroused the interest of many researchers.

#### 3.3.1. Mechanism of Action of Phenformin

**Figure 4:**
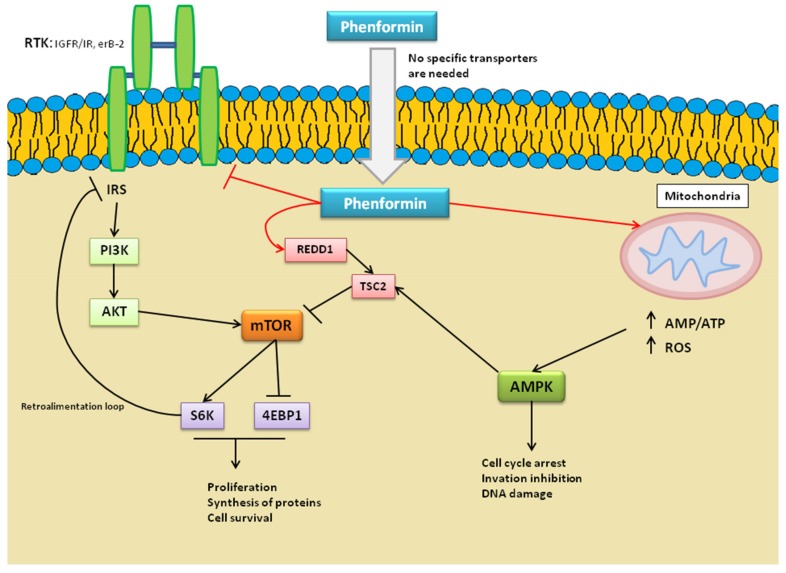
**Mechanism of Action of Phenformin.** When phenformin passes freely through the cellular membrane, it acts in three different ways: 1) inhibition of the complex I of the mitochondrial respiratory chain, 2) activation of REDD1 protein, 3) inhibition of insulin receptor substrate (IRS) receptors. These actions lead to AMP-activated protein kinase with energy detection (AMPK) activation and mTOR pathway blocking by the activation of its inhibitor and the retro-alimentation loop block. This process leads to cell cycle arrest, invasion inhibition, and DNA damage. It also blocks proliferation, synthesis of proteins, and cell survival. ROS, reactive oxygen species; RTK, receptor tyrosine kinase; IGFRs, insulin-like growth factors; EGFRs, epidermal growth factor receptors; IRS, insulin receptor substrate.

As shown in the Figure 4, the mechanism of action of phenformin is very similar to metformin, leading to similar effects. However, the effect of phenformin is more potent than metformin due to the way it enters into the cells. It is known that metformin is a very hydrophobic compound and requires organic cation transporters (OCTs) to pass through the cellular membrane [47]. In contrast, phenformin does not need any transport protein to be capable of entering in an uninhibited way [48]. This fact is relevant for two reasons: it not only permits a higher phenformin concentration inside tumor cells but also achieves successful treatment in tumors with no OCT overexpression.

Studies have shown that AMP-activated protein kinase with energy detection (AMPK) is an important cell target of biguanides, since it is activated in the liver in response to therapeutic doses of these drugs. This is the key direct effect of phenformin on the cancer cells. The AMPK activation occurs due to the inhibition of the complex I of the mitochondrial respiratory chain, thus increasing the cellular AMP (and ADP) to ATP ratio and the subsequent overproduction of reactive oxygen species (ROS) [48]. The production of ROS in itself constitutes a death effect on tumor cells.

On the other hand, AMPK inhibition leads to the inhibition of mTOR pathways, a key regulator of cell proliferation [49,50]. This kinase activates the inhibitor of mTOR, TSC2 protein. Then, TSC2 inhibits mTOR by capturing the Rheb protein, and this leads to S6K protein inhibition and 4E-BP1 protein activation, a transcription initiation factor [49,50]. This way, the tumor cellular growth decreases.

Furthermore, the TSC/Rheb/mTOR/S6K pathway also regulates the insulin receptor substrate (IRS) that constitutes an important retro-alimentation loop [51]. S6K blocking leads to the activation of several proteins upstream, including IR/IGF1R (insulin-like growth factor 1) and AKT. This fact produces the activation of cellular proliferation that normally occurs with the rapamycin inhibitors. Surprisingly, it has been demonstrated that phenformin is able to block this phenomenon, decreasing the IR/IGF1R transduction [52]. Guo et al (2017) demonstrated this fact through a western assay, which showed that it reduces the expression of several proteins downstream, such as ErbB2, AKT, ERK, and mTOR. In this way, phenformin shows better results than the classical rapamycin inhibitors [52].

Phenformin has more advantages in comparison to rapamycin inhibitors. On the one hand, it avoids the hyperglycemias produced by those types of inhibitors that improve tumor growth in patients [53]. On the other hand, it has been demonstrated that rapamycin inhibitors improve mitochondrial efficiency, giving an enormous surveillance power to cancer stem cells (CSCs) [48]. So it could be said that phenformin use helps to fight against CSCs in an indirect way. This evidence, added to the fact that the PI3K/mTOR pathway is one of the most important processes for CSC surveillance, makes phenformin an interesting anti-CSC treatment. In fact, there are studies that have demonstrated the ability of phenformin to decrease CSC selective markers. In contrast, metformin seems to have no effect on these [54].

Pathways that regulate the epithelial mesenquimal transition (EMT), such as receptor tyrosine kinase (RTK), tumor growth factor-β (TGF-β)/Smad, Notch, and Wnt signaling pathways are anticancer drug targets. Particularly, the RTKs involved in the EMT process consist of epidermal growth factor receptors (EGFRs) and insulin-like growth factors (IGFRs). As described before, phenformin has the ability to block this pathway.

In relation to cellular cycle arrest, Jackson et al. (2017) [49] studied phenformin’s effect on SKOV3, IGROV-1, and Hey cell lines. Phenformin inhibits both CDK4 and D glycine and activates p21 proteins. They also carried out an Annexin V assay, which showed that the phenformin treatment induces G0/G1 cell cycle arrest and decreases S phase in a dose-dependent manner.

#### 3.3.2. Phenformin in Cancer Treatment

The discovery that diabetic patients treated with biguanides have a low risk of suffering from cancer has caused an interest in their use as antitumor treatments [55,56]. Compared to metformin, phenformin may have a higher antitumor effectiveness, due to its greater absorption by tumor cells and its higher potency and tissue bioavailability [57]. Studies of several tumor types (e.g., breast, lung, glioblastoma, colon, melanoma, and prostate cancer) demonstrate that phenformin is more potent in inhibiting cell proliferation and tumor growth than metformin, both in vitro and in vivo [57].

#### Phenformin Effect on Differentiated Cancer Cells

In breast cancer, the potential of phenformin as an antineoplastic agent more potent than metformin has been demonstrated in chemoprevention and in the treatment of both estrogen receptor (ER)-positive MCF-7 and triple negative receptor MDAMB231 in xenografts in vivo [58]. Moreover, the half maximal effective concentration (EC50) of phenformin for inducing cell death in a model of HPV+head and neck cancer was 840 times lower than that of metformin [59]. Analogous results were obtained in the treatment of cholangiocarcinoma (CCA), an aggressive malignancy extremely resistant to chemotherapeutic agents. Thus, phenformin inhibits CCA cell growth both in vitro and in vivo. It also induces apoptosis and autophagy of CCA cells [60]. In lung tumors, it has been proven that phenformin has a greater bioavailability and is able to induce more potent stress energy than metformin [61]. Phenformin could have a possible use for early-stage lung tumors or as adjuvant therapy after resection of non-small cell lung cancer. However, in advanced stages of the disease, it may be synergized with other forms since phenformin as an anticancer agent alone is ill-suited as a therapy [62]. In ovarian cancer (OC), although phenformin has antitumor effects, as previously demonstrated with metformin [63,64], it has not yet been determined whether it is better. Phenformin inhibited cell proliferation and induced G1 cell cycle arrest and apoptosis [49]. In melanoma, a recent study has shown that unlike metformin, phenformin strongly reduces the viability, growth, and invasion of melanoma cells by inducing apoptosis in both two-dimensional and three-dimensional models (spheroids) [54]. However, the combination of phenformin with other chemotherapeutic agents has shown better therapeutic results than the use of phenformin alone [54]. Until now, there is only one single clinical trial in phase I, being carrying out by Paul Chapman from MD Memorial Sloan Kettering Cancer Center (NCT03026517). This study was published for the first time on ClinicalTrial.org on 20 January 2017 and has an estimated duration of 2 years. It is aimed at testing the safety of the use of phenformin in combination with standard chemotherapy. A dabrafenib plus trametinib combination is a standard treatment for patients with metastatic melanoma with *BRAF* gene mutation [65]. In the dose escalation phase, both patients who have already been treated with a *BRAF* and/or *MEK* inhibitor and treatment-naïve patients will be eligible. Moreover, cohorts of patients will be treated with standard-dose dabrafenib (150 mg PO BID) plus trametinib (2 mg PO QD) and increasing doses of phenformin. The results of this pilot clinical trial will undoubtedly be crucial for the future application of phenformin as an antitumor agent.

#### Phenformin’s Effect on Cancer Stem Cells

Even after the best available treatments for primary tumors, cancer may reappear after an extended disease-free interval. Through that period, cancer cells remain inactive at primary or metastatic sites, evading adjuvant cytotoxic treatments [66]. Nowadays, we know of the existence of a subpopulation of cells named cancer stem cells (CSCs), characterized by a quiescent profile but able to proliferate and self-renew under certain conditions [67]. CSCs are very resistant to treatments that target rapidly dividing tumor cells. Different studies have determined that most antitumor drugs are not effective against cancer stem cells due to their drug-resistance, causing relapse and metastasis [68]. Recent evidence has shown that CSCs show a different metabolic profile compared to the population of bulk tumors: a promising antitumor strategy is to point to precise metabolic pathways that drive CSC behavior. The modulation of cell metabolism by inhibiting the mitochondrial-1 complex obtained promising results in different cancers, including melanoma [69]. Phenformin is among the few anticancer drugs capable of targeting cells with the high phenotype of CSC aldehyde dehydrogenase (ALDH), although not specifically. Studies have shown that they inhibit the mitochondrial complex 1, indicating that their metabolic effects vary according to the stage of cellular transformation, strongly depleting nucleotide triphosphates (NTP), and may prevent the synthesis of nucleotides. In addition, resistance to single and combined therapies in melanoma cancer is frequently related to positive regulation of oxidative phosphorylation (OXPHOS) [70]. Moreover, CSCs often overexpress genes associated with the beta-oxidation of fatty acids, OXPHOS, and glucose uptake [71]. Thus, phenformin, as an OXPHOS inhibitor able to target both non-CSCs and CSCs, could be an effective strategy in melanoma therapy [69]. Furthermore, phenformin inhibits the self-renewal of glioblastoma stem cells (GSCs) and induces cell death at higher concentrations. Moreover, GSCs seem to be much more sensitive to phenformin than glioma cells and showed more growth arrest and induction of apoptosis both in vitro and in vivo [72]. In addition, the effect of phenformin on GSCs is mediated by the upregulation of let-7, miR-137, and miR-124 [73] and the downregulation of HMGA2 [72]. Phenformin had a similar effect on lung cancer stem cells (side population cells, i.e., SP cells) [74] and breast cancer stem cells [57].

#### Combined Therapy Using Phenformin

Cancer therapy in which two or more therapeutic agents are combined improves efficacy compared to the monotherapy approach. Combined therapy potentially reduces drug resistance and provides therapeutic benefits against cancer, such as reduced growth tumor and metastatic potential, the reduction of populations of cancer stem cells, and the induction of apoptosis [75].

Different studies have shown that the combination of phenformin and other anticancer agents would be a good strategy in the treatment of some types of cancer.

Phenformin and oxamate/dichloroacetate (DCA) is a promising therapeutic combination against cancer. Studies have shown that tumor cells have metabolic flexibility for the generation of ATP by selecting either oxidative phosphorylation (OXPHOS) or glycolysis, depending on the disposal of nutrients in the tumor microenvironment. In this context, it is known that phenformin induces apoptosis and inhibits cell proliferation under glucose restrictive conditions by the inhibition of complex I in mitochondria and the subsequent overproduction of reactive oxygen species (ROS) [59]. In addition, DCA or oxamate, used individually, inhibit the LDH activity and the production of lactate by the cells, producing OXPHOS activation and subsequent ATP generation. However, this induces cell proliferation inhibition without cell death [76]. Recent studies have demonstrated, in a syngeneic mouse model using several tumor cell lines, that a phenformin/oxamate combination is more effective than a phenformin/LDH knockdown combination, inducing a reduced tumor size and increased apoptosis compared to controls [59]. The blockage of both complex I and LDH may be lethal for tumor cells, inducing a metabolic catastrophe due to cellular ATP pool depletion. Phenformin decreases the activation of OXPHOS, and the DCA or oxamate decreases glycolysis. This leads to apoptosis initiation, depression of tumor growth, and cell death [59,77]. Moreover, DCA, an inhibitor of the enzyme pyruvate dehydrogenase kinase glycolysis, which decreases lactic acidosis induced by biguanides, potentiates the effects of phenformin on the induction of cell death in GSCs and prolongs the survival of mice carrying xenografts, in comparison to each treatment alone [72].

On the other hand, most patients with mutant BRAF melanoma develop drug resistance during their therapy. The treatment with vemurafenib, an inhibitor of BRAF protein kinase, has shown remarkable antitumor activity; however, many of the patients developed resistance [78]. In this context, preclinical studies suggest a phenformin and BRAF inhibitor combination may be more effective than a single-agent BRAF inhibitor to treat patients with BRAF-mutated melanoma [79]. Indeed, the antitumor effects of the combination of phenformin with PLX4720, a BRAF inhibitor, on the proliferation of BRAF-mutated melanoma both in vitro and on tumor growth promoted by BRAF in vivo have been investigated. The combination of phenformin with BRAFi (BRAF inhibitor) results in improved efficacy in both cell culture and animal models. This is due to the ability of this combination to suppress the JARID1B-positive subset of tumor cell proliferation, which is resistant to single-agent therapy. In addition, BRAFi seems to target the population of JARID1B-negative melanoma cells, the cells most present in the tumors, and avoids the population in the positive slowdown cycle of JAR1D1B [80]. Currently, the only clinical trial using phenformin is underway, to assess the effect of this biguanide and its combination with different chemotherapeutic and biological drugs (NCT03026517). The clinical trial carried out at the Weill Medical College of Cornell University with the Massachusetts General Hospital focused on assessing the safety of phenformin administration with dabrafenib+trametinib (standard combination) in patients with mutated melanoma BRAFV600E/K. The combination of dabrafenib plus trametinib is a standard treatment for patients with BRAF-mutated melanoma [65]. This study has not yet been completed, so far meeting phase I.

Another combination could be the use of phenformin and extracellular signal-regulated kinase inhibitors (ERKs). In the treatment of mutant melanoma NF1, preclinical studies suggest that kinase inhibitors regulated by extracellular signal (ERK) are likely to provide benefits, although with limited efficacy as single agents [81]. In this context, Trousil and collaborators demonstrated that combined treatment with SCH772984 and phenformin could be a promising therapeutic option. In fact, phenformin and SCH772984 synergistically inhibited proliferation in NF1-mutant cancer cells and cooperatively induced apoptosis [82]. It also promotes the expansion of subtype H3K4 demethylase KDM5B-positive melanoma cells, which are slow cycling and treatment resisting. On the other hand, another study has shown that the combination of both drugs can completely suppress mTOR signaling, a known effector of NF1 loss. The treatment with SCH772984 induces an oxidative metabolic program increasing PGC1alpha expression and the rate of oxygen consumption, which indicates that these cells were based more on the oxidative phosphorylation after treatment [83].

Finally, phenformin and temozolomide combined therapy in glioblastoma treatment could be an interesting strategy. Today, glioblastoma treatment focuses on the combination of radiotherapy with temozolomide (TMZ), which has achieved an increase in survival in phase III clinical trials [84]. Temozolomide, a methylating agent, is able, at physiological pH, to hydrolyze spontaneously to its active metabolite 3-methyl-(triazen-1-yl) imidazole-4-carboxamide (MTIC). Therefore, the acidic extracellular pH in tumors might decrease temozolomide efficacy, reducing its spontaneous conversion. To try to solve this problem, Jiang et al. have experimented with a combination therapy of TMZ with phenformin. Their results have demonstrated that the combined treatment induces a synergistic effect on glioblastoma stem cell (GSC) death both in vitro and in vivo, improving the antitumor effects of phenformin and decreasing the concentrations of this drug, in this way allowing the enhancement of its efficacy and safety [67].

## 4. Current Challenges and Future Perspectives

More and more epidemiological studies seem to relate cancer to diabetes, constructing an increasingly stronger relationship between both diseases, without drawing any conclusions from data yet.

What can be related is the biochemical link between both diseases, chronic hyperinsulinemia (responsible for insulin resistance), the pro-inflammatory environment, and the greater bioavailability of sex hormones. There is a statistical relationship between diabetes and cancer, and there are more and more observational epidemiological studies that strengthen it. Although this relationship is getting stronger, conclusions cannot be drawn from the data. This research area is quite important, but so far the mechanism of action of biguanides, regarding their role as antineoplastics, has not been elucidated completely, causing new lines of research to open in this regard. It is fundamental, when considering tumor cell metabolism in cancer therapy, to understand the direct biochemical relationship between pathologies, cancer, and diabetes, not only with drugs from this family of compounds but also with other drugs that decrease insulin levels, such as the glitazones. Therefore, it is not a matter of lowering glucose levels for tumor cells to slow down their growth, since the use of drugs that would fulfill this function, such as sulfonylureas, increase pancreatic insulin secretion and are associated with a higher incidence of cancer. New research that allows us to understand the molecular mechanisms that connect diabetes type 2 and its treatment with cancer is fundamental for us to establish primary prevention strategies, early detection, and possible effective combinatorial therapies with antineoplastic drugs. Lactic acidosis, although more frequent in hematological malignancies, has also been described in solid tumors, mainly breast, lung, and colon, generally associated with significant hepatic involvement. While it is true that phenformin is a drug that was withdrawn from the market due to lactic acidosis and that cancer patients present metabolic acidosis with gap anion raised by increased production or decreased lactic acid clearance, it seems logical to think that the dosage for therapy combined with antineoplastic drugs should be lower than the doses that were known for those antidiabetics that were withdrawn due to cases of lactic acidosis mortality. Briefly, the presence of lactic acidosis in the oncological patient is the manifestation of a serious metabolic problem that indicates the severity of tissue hypoxia.

It is well known that metformin is nontoxic and might be extremely useful for enhancing the treatment efficacy of mechanism-based and biologically targeted drugs [14] and does not cause hypoglycemia in non-diabetic patients [85]. Compared to metformin, phenformin may have a higher antitumor effectiveness due to its greater absorption by tumor cells and its higher potency and tissue bioavailability [57]. Based on the review of the available evidence, most knowledge reported about phenformin is its ability to inhibit several processes in relation to tumor development and growth. Key components of its antiproliferative effects are the induction of apoptosis, cellular stress, cell cycle G1 arrest, and epithelial mesenquimal transition (EMT) inhibition [48,49,52,58]. Moreover, phenformin is among the few anticancer drugs capable of targeting CSCs from different types of tumors such as breast cancer [57], melanoma [69], and lung cancer [74], among others. In addition, different studies have shown that the combination of phenformin and other anticancer agents would be a good strategy in the treatment of some types of cancer [59,77,78,79]. This combinational therapy can reduce the doses of phenformin and therefore decrease the lactic acidosis that it produces. All these studies justify the need for more preclinical and clinical trials to confirm the potential antineoplastic activity of phenformin and to determine therapeutic doses and safety in non-diabetic patients. Currently, there is only one clinical trial focusing on phenformin and its combination with different chemotherapeutic and biological drugs (NCT03026517). This study has not yet completed phase I, and its results undoubtedly will be of great interest in determining the antitumor role of phenformin. Thus, we need to develop many more clinical trials to determine the therapeutic dose of phenformin as an antitumor agent. Moreover, it would be of great interest to perform a clinical trial, similar to the one being developed for metformin, with non-diabetic patients, but in this case focused on phenformin (NCT03772964). This could provide valuable data on the safety of the use of phenformin. Hence, much work remains to be done, both in preclinical and clinical stages, to elucidate the real possibility of using this biguanide as an antitumor agent and to be able to affirm, decisively, the lower risk of cancer under biguanide treatment.
